# Integrated machine learning for modeling bearing capacity of shallow foundations

**DOI:** 10.1038/s41598-024-58534-5

**Published:** 2024-04-09

**Authors:** Yuzhen Liu, Yan Liang

**Affiliations:** 1 Bim School of Technology and Industry, Changchun Institute of Technology, Changchun, 130012 Jilin China; 2 Infrastructure Logistics Office, Jilin Engineering Normal University, Changchun, 130012 Jilin China

**Keywords:** Geotechnical engineering, Bearing capacity analysis, Machine learning, Metaheuristic algorithms, Engineering, Mathematics and computing

## Abstract

Analyzing the stability of footings is a significant step in civil/geotechnical engineering projects. In this work, two novel predictive tools are suggested based on an artificial neural network (ANN) to analyze the bearing capacity of a footing installed on a two-layered soil mass. To this end, backtracking search algorithm (BSA) and equilibrium optimizer (EO) are employed to train the ANN for approximating the stability value (SV) of the system. After executing a set of finite element analyses, the settlement values lower/higher than 5 cm are considered to indicate the stability/failure of the system. The results demonstrated the efficiency of these algorithms in fulfilling the assigned task. In detail, the training error of the ANN (in terms of root mean square error—RMSE)) dropped from 0.3585 to 0.3165 (11.72%) and 0.2959 (17.46%) by applying the BSA and EO, respectively. Moreover, the prediction accuracy of the ANN climbed from 93.7 to 94.3% and 94.1% (in terms of area under the receiving operating characteristics curve—AUROC). A comparison between the elite complexities of these algorithms showed that the EO enjoys a larger accuracy, while BSA is a more time-effective optimizer. Lastly, an explicit mathematical formula is derived from the EO-ANN model to be conveniently used in predicting the SV.

## Introduction

A significant portion of recent literature highlights the positive effect of newly developed technology on diverse engineering domains^1–5^. These developments include new approaches, devices, and software that assist experts towards facilitating data acquisition, simulation, and eventually solving complex engineering problems^6–10^. Taking image processing as an evident example of these sophisticated techniques, Jafarzadeh et al.^11^ used it along with experimental data to validate their analytical model for simulating flexible circular submerged mound motion in gravity waves. Advanced sensors^12^, artificial intelligence models^13^, 2D and 3D analytical software^14,15^, and innovative materials^16^ are other examples that have been promisingly used.

Focusing on civil and geotechnical engineering, many sub-disciplines often benefit from leading methods/tools e.g., in structural and material analysis^17–21^. Rock, soil, concrete, and composite materials are eminent examples of this statement^22–26^. Among these research hotspots, geotechnical and environmental issues have received significant attention, particularly when it comes to investigating project sites, designing elements, and material analysis using sophisticated techniques^27–31^. For instance, Khalil et al.^32^ employed self-potential, electrical resistivity tomography, and frequency domain electromagnetic methods to successfully map an under-hazard gypsum mine. In another study by Khoei et al.^33^, an extended finite element method was employed to model the density-driven flow in heterogeneous porous media having micro- and macro-fractures. Jafarzadeh et al.^34^ used an experimental approach and proposed an innovative flexible breakwater for wave control in shallow water.

Footings are crucial geotechnical elements whose proper design on horizontal soil surfaces is a very significant step in civil/geotechnical engineering projects^35,36^. It is dependent on two groups of properties belonging to (1) footing (e.g., width, depth, and shape) and (b) soil (e.g., shear strength and unit weight). The safety factor and acceptable settlement are two principal measures that need to be considered to determine the allowable bearing pressures^37^. As is known, analyzing the bearing capacity has been a nonlinear and complex challenge for engineers because of the influence of various parameters on it^38,39^. Hence, machine learning models are known as one of the most efficient approaches (amongst various evaluative techniques like upper bound^40^ and lower bound^41^ finite element limit analysis) for exploring such parameters in a fast, yet inexpensive, way. Due to its many advantages, the use of intelligent models has been very popular in geotechnical engineering^42–45^ and many other fields^46,47^.

Artificial neural networks (ANNs), as well as support vector-based^48^, tree-based^49^, and fuzzy models, are one of the most capable notions of machine learning methods used for various prediction purposes^50–52^. Many scholars have benefitted ANNs for predicting various geotechnical parameters like bearing capacity^53–55^. Shahin et al.^56^ employed an ANN to forecast the settlement of shallow foundations settled on cohesionless soils. Debnath and Dey^57^ suggested the use of a well-known intelligent model called support vector regression developed with exponential radial basis kernel function (SVR-ERBF) for estimating the bearing capacity of stone columns reinforced by geogrid. Pham et al.^58^ investigated the applicability of ANN and random forest (RF) for estimating the pile axial bearing capacity. They concluded that these intelligent tools outperform empirical approaches. Also, the RF achieved a lower root mean square error (RMSE) in the prediction phase in comparison with ANN (98.161 vs. 116.366). Acharyya et al.^37^ could successfully (with correlations higher than 99%) predict the bearing capacity of a square footing using ANN. The data was provided through a 3D finite element analysis in the Plaxis environment. A comparison between well-known predictive models, namely RF regression, ANN, M5P model tree, support vector machine (SVM) with polykernel and RBF kernel functions for simulating the ultimate bearing capacity of foundations settled on rock surfaces was conducted by Dutta et al.^59^. It was shown that RF regression and SVM-RBF present the most accurate prediction of the intended parameter. More studies about hiring conventional intelligent model can be found in earlier literature^60–63^.

Metaheuristic-integrated schemes have shown high promise for engineering optimization tasks^64–66^. Ghanizadeh et al.^67^, for instance, proposed a multivariate adaptive regression splines (MARS) model that was optimized by escaping bird search (EBS) algorithm for developing a hybrid bearing capacity evaluator for geogrid-reinforced sandy bed on vertical stone columns in soft clay. Alzabeebee^68^ developed a new explicit equation based on genetic algorithm multi-objective evolutionary regression analysis for undrained bearing capacity estimation of footing settled on aggregate pier reinforced terrain. Moayedi et al.^69^ examined the efficiency of four popular metaheuristic optimizers including ant colony optimization (ACO), whale optimization algorithm, moth–flame optimization, and league champion optimization applied to an ANN for predicting the bearing capacity. The findings of this research showed that these algorithms can properly assist the ANN in learning the nonlinear pattern of bearing capacity. The ACO-ANN, with 96.5 and 94.4% accuracy in the training and testing phase, respectively, emerged as the most reliable predictor. Likewise, a comparison between dragonfly algorithm (DA) and Harris hawks optimization (HHO) was conducted by Moayedi et al.^70^. The prediction accuracies of 0.942 and 0.915 for the hybrid models versus 0.89 obtained for the conventional ANN indicated (a) the improvements resulted from the functioning of the DA and HHO and (b) the higher optimization capability of the DA. Harandizadeh and Toufigh^71^ used a combination of neural-fuzzy (NF) system and group method of data handling (GMDH) improved by the PSO and gravitational search algorithm for simulating the axial bearing capacity of driven piles. Based on the calculated RMSE (1375 and 1740.7 for the PSO-NF-GMDH and NF-GMDH, respectively), the competency of the PSO technique was deduced.

Regarding the promising performance of metaheuristic techniques (as explained in the above literature), it is necessary to update the engineering simulations with the new generation of these algorithms. Therefore, this study develops and evaluates two novel integrative models, based on backtracking search algorithm (BSA) and equilibrium optimizer (EO) used for fine-tuning the weights and biases of an ANN model. These algorithms are based on metaheuristic search schemes that find the optimal solution to any given numerical problem.

Although popular optimizers (like PSO^72^, ACO^73^, and imperialist competitive algorithm (ICA)^74^) have been widely used for bearing capacity calculation, to the best knowledge of the author, no former study has benefitted from the BSA and EO for this objective. According to the relevant literature, these two algorithms possess high optimization competency for dealing with machine learning models^75,76^. Notably, a conventional ANN is also considered as the comparative benchmark model to examine the enhancement of prediction achieved by the suggested algorithms. The explicit numerical expression of the used models is also presented in the last part to attain a predictive formula for future bearing capacity analysis. Moreover, the used data will be exposed to the principal component analysis (PCA) method for addressing the importance of key factors that influence bearing capacity.

## Methodology

### Backtracking search optimization algorithm

One of the most potent optimization techniques is the backtracking search that is designed by Civicioglu^77^. The BSA benefits both local exploitation and global exploration options. It indicates that the algorithm first uses the whole space to seek the solution and also checks the vicinity of the found solution for the best options^78^.

The BSA goes through five major stages including initialization, selection-I, mutation, crossover, and selection-II to find the solution to the given problem. The first step, like any other population-based technique, is to generate a random population over the search space. To do so, the BSA uses a uniform distribution function (U) as follows:

1 $${P}_{i, j}\backsim U\left({low}_{j}, {up}_{j}\right),\, \mathrm{i }= 1, 2, \dots ,\mathrm{ N},\,\mathrm{ j }= 1, 2, \dots ,\mathrm{ D}$$

in which, the lower and upper bounds of the given problem are represented by ${low}_{j}$ and ${up}_{j}$, ${P}_{i}$ stands for the position of the i^th^ member, D shows the dimension, and N denotes the population size.

In the first selection process, let a and b be uniform real values that may vary from 0 to 1. Then Eqs. 2 and 3 are used for determining the search direction and redesigning the old members, respectively.

2 $${oldP}_{i, j}\backsim U({low}_{j}, {up}_{j})$$

3 $$if \,a<b, then\,odlP :=P | a, b\backsim U(0, 1)$$

Next, the order of the members is randomly shuffled by the below function:

4 $$odlP :=\mathrm{permuting }\,(odlP)$$

For doing the third step (i.e., mutation), Eq. 5 expresses the way that the mutant members are formed:

5 $$Mutant ={\text{P}}+{\text{F}}. (odlP-P)$$

In the above relationship, F is a parameter used to control the step size amplification of the search direction.

The fourth step of the algorithm (i.e., crossover) is devoted to setting the final form of the trial population T. Here, applying a restriction strategy is of high importance because some of the members may trespass the boundaries of the problem space.

Lastly, the algorithm ends up with the second selection stage. In this stage, the algorithm updates the values of ${P}_{i}$. It is implemented by using a greedy selection process that is based on preferring ${T}_{i}$ s with higher fitness values. Once the fitness value of the most successful member (${P}_{best}$) is better than the global minimum, this member is considered as the global minimizer. Also, the global minimum value is updated to the fitness value of ${P}_{best}$^79^.

### Equilibrium optimizer

Metaheuristic algorithms are mostly inspired by the foraging/social behavior of animals in nature. But the essences of the EO algorithm are laws in physics. Faramarzi et al.^80^ designed this algorithm in 2020 for dealing with optimization problems^81^. Three main steps of the EO are initialization, equilibrium pool of candidates, and updating the concentration. Mathematical explanations of these steps are presented here. Initially, the particles are generated. Each of which contains a response to the given problem with a concentration vector (CV). Assuming n as the number of individuals, and ${c}_{max}$ and ${c}_{min}$ as the upper bound and lower bounds of the problem space, Eq. 6 expresses the random creation of the CV:

6 $${\overrightarrow{V}}_{i}={c}_{min}+\left({c}_{max}- {c}_{min}\right)\hspace{0.17em}\times \hspace{0.17em}r\mathrm{ \,i}\hspace{0.17em}=\hspace{0.17em}0, 1, \dots ,\mathrm{ n}$$

Where *r* stands for a random value ranging in [0,1].

Like any other optimization technique which pursues a strategic objective (e.g., finding the best nectar in artificial bee colony algorithm), the aim of the EO is to reach an equilibrium state for the complex. The optimal solution may emerge once the purpose state is met. In the EO, the four most successful agents of the population are assigned for this purpose. This is because the algorithm cannot find out about the level of concentration in which the optimization is achieved. A fifth agent is also considered. It represents the average of the four selected agents. The first four agents help the EO to benefit from a better diversification ability, while the fifth one acts toward better exploitation. These agents are stored in a vector called an equilibrium pool:

7 $${\overrightarrow{P}}_{eq, pool}=\left[{\overrightarrow{P}}_{eq(1)}, {\overrightarrow{P}}_{eq(2)}, {\overrightarrow{P}}_{eq(3)}, {\overrightarrow{P}}_{eq(4)}, {\overrightarrow{P}}_{eq(mean)}\right]$$

The third step is called updating the concentration in which a plausible balance is aimed between diversification and intensification. We have:

8 $$\overrightarrow{F}={e}^{-\overrightarrow{\sigma }(t - {t}_{0})}$$

where $\overrightarrow{\sigma }$ represents a random vector between 0 and 1. The parameter t decreases as the iteration increases:

9 $$t = \left( {1 - \frac{{it}}{{t_{{max}} }}} \right)^{{\left( {\alpha \left( {\frac{{it}}{{t_{{max}} }}} \right)} \right)}}$$

where $\alpha$ is a constant value used to control the intensification capability. Also, ${t}_{max}$ and *it* stand for the maximum and current iterations, respectively. In addition, the EO uses *β* to improve the diversification and intensification processes as follows:

10 $${\overrightarrow{t}}_{0}=\frac{1}{\overrightarrow{\sigma }}{\text{ln}}\left(-\beta sign\left(\overrightarrow{r}-0.5\right)\left[1-{e}^{-\overrightarrow{\sigma }t}\right]\right)+t$$

According to the rules, the value of *β* is directly proportional to the quality of diversification, and adversely proportional to the quality of intensification. Generation rate (R) is another parameter that is defined to improve the intensification agent. It is expressed by Eq. 11:

11 $$\overrightarrow{R}={\overrightarrow{R}}_{0} {e}^{-\overrightarrow{\sigma }(t-{t}_{0})}$$

12 $${\overrightarrow{R}}_{0}=\overrightarrow{RCP} ({\overrightarrow{c}}_{eq}- \overrightarrow{\sigma } \overrightarrow{C})$$

13 $$\overrightarrow {{RCP}} = \left\{ {\begin{array}{*{20}l} {0.5r_{1} } \hfill & {r_{2} { > }RP} \hfill \\ 0 \hfill & {otherwise} \hfill \\ \end{array} } \right.$$

in the above relationships, ${\overrightarrow{R}}_{0}$ is the initial value, ${r}_{1}$ and ${r}_{2}$ symbolize random values that may vary from 0 to 1. Also, regarding a probability value *RP*, the term $\overrightarrow{RCP}$ is used to determine whether the generation rate is applied to the updating process. Eventually, Eq. 14 gives the updating relationship of the EO algorithm:

14 $$\overrightarrow{C}={\overrightarrow{c}}_{eq}+\left(\overrightarrow{C}-{\overrightarrow{c}}_{eq}\right) \overrightarrow{F}+\frac{\overrightarrow{R}}{\overrightarrow{\sigma } V}(1- \overrightarrow{F})$$

where V = 1^82^.

## Data collection

In order to propose an intelligent model for any engineering problem, the models should be fed by a set of relevant data. in the case of this study, this data is obtained from a series of finite element analyses. The feasibility of this method for various geotechnical purposes has been broadly investigated in earlier literature e.g., modeling mass transport problem in fractured porous media^83^.

A two-layered soil which bears a shallow footing is selected as the system. This system was analyzed under 2D axisymmetric circumstances. About the meshing, the system was modeled via 15-node triangular elements where the Mohr–Coulomb model is considered for the material. Further details for the performed FEM analysis can be found in similar reference studies such as^84^. The system had seven variables, namely friction angle, dilation angle, unit weight (kN/m^3^), elastic modulus (kN/m^2^), Poisson's ratio (v), setback distance, and applied stress (kN/m). The objective of the FEM analysis was to measure settlement values. Considering different combinations of these variables, a total of 901 different stages were run and the settlement was obtained. Table 1 shows some examples of the produced dataset.

**Table 1:** An example of the FEM analysis results.

Features	Sample number									
	1	2	3	4	5	6	7	8	9	10
Friction angle	30.00	30.00	33.00	33.00	36.00	36.00	39.00	39.00	42.00	42.00
Dilation angle	3.40	3.40	5.80	5.80	8.00	8.00	10.00	10.00	11.50	11.50
Unit weight (kN/m^3^)	19.00	19.00	19.90	19.90	20.50	20.50	20.90	20.90	21.10	21.10
Elastic modulus (kN/m^2^)	17,500.00	17,500.00	25,000.00	25,000.00	35,000.00	35,000.00	50,000.00	50,000.00	65,000.00	65,000.00
Poisson's ratio (v)	0.33	0.33	0.31	0.31	0.29	0.29	0.27	0.27	0.25	0.25
Setback distance	1.00	5.00	7.00	5.00	5.00	5.00	3.00	7.00	3.00	7.00
Applied stress (kN/m)	99.08	271.27	189.06	261.48	80.09	353.79	527.95	761.30	65.60	965.08
Settlement (m)	0.01	0.08	0.02	0.10	0.00	0.05	0.06	0.09	0.00	0.06

**Figure 1:**
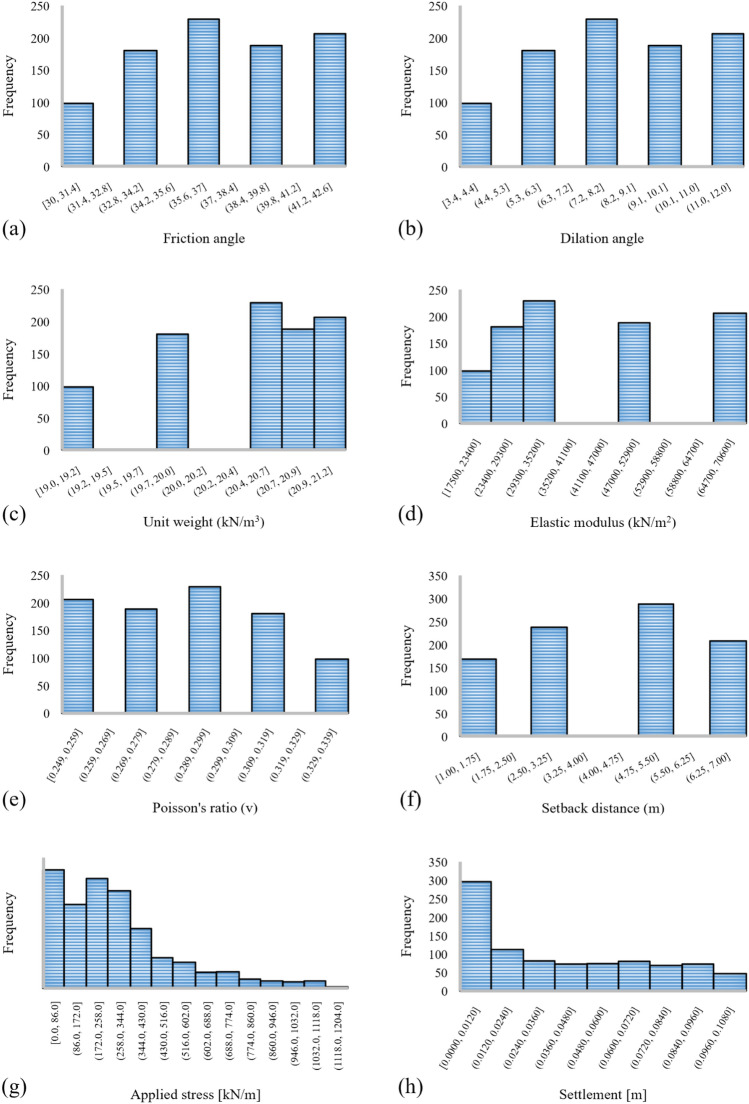
The histogram of the dataset inputs and target.

After extracting the settlements, they are listed along with the mentioned variables for each run, and accordingly, a dataset with seven inputs and one target is created. Table 2 statistically describes the used dataset. Also, an illustration of the histogram charts of the mentioned parameters is shown in Fig. 1.

**Table 2:** Descriptive statistics of the dataset.

Features	Symbol	Descriptive index						
		Mean	Standard error	Standard deviation	Sample variance	Skewness	Minimum	Maximum
Friction angle	FA	36.746	0.130	3.909	15.283	− 0.142	30.000	42.000
Dilation angle	DA	8.278	0.087	2.614	6.834	− 0.390	3.400	11.500
Unit weight (kN/m^3^)	UW	20.438	0.022	0.655	0.429	− 0.954	19.000	21.100
Elastic modulus (kN/m^2^)	EM	41,087.680	546.654	16,408.723	269,246,192.502	0.217	17,500.000	65,000.000
Poisson's ratio (v)	PR	0.286	0.001	0.028	0.001	0.138	0.249	0.333
Setback distance	SD	4.190	0.069	2.075	4.307	− 0.135	1.000	7.000
Applied stress (kN/m)	AS	289.739	7.895	236.966	56,152.918	1.264	0.000	1132.654
Settlement (m)	–	0.038	0.001	0.033	0.001	0.464	0.000	0.100

The recorded values of settlement vary from 0 to 10 cm. The settlements lower than 5 cm are the indications of a stable system, while the failure of the system is represented by the settlements larger than 5 cm. The purpose of this classification is to train the networks for calculating the stability value (SV) instead of settlement. This settlement threshold is a well-accepted value among the experts for detecting the failure in soil-foundation systems; and it has been earlier considered in similar studies such as^85,86^.

In order to determine the training and testing data (used for inferring and generalizing the SV pattern), a random division is carried out. Devoting 80% of data to the training phase, 721 samples are selected for analyzing the relationship between the SV and input parameters. Next, the rest of the data (i.e., 180 samples) are used as new conditions of systems to evaluate the prediction robustness of the developed models.

## Results and discussion

The overall aim of this study is to present two novel predictive models for approximating the failure/stability of soil-foundation systems through bearing capacity analysis. The findings are presented and discussed in this part. The proposed models are ensembles of metaheuristic techniques with a popular neural computing tool.

**Figure 2:**
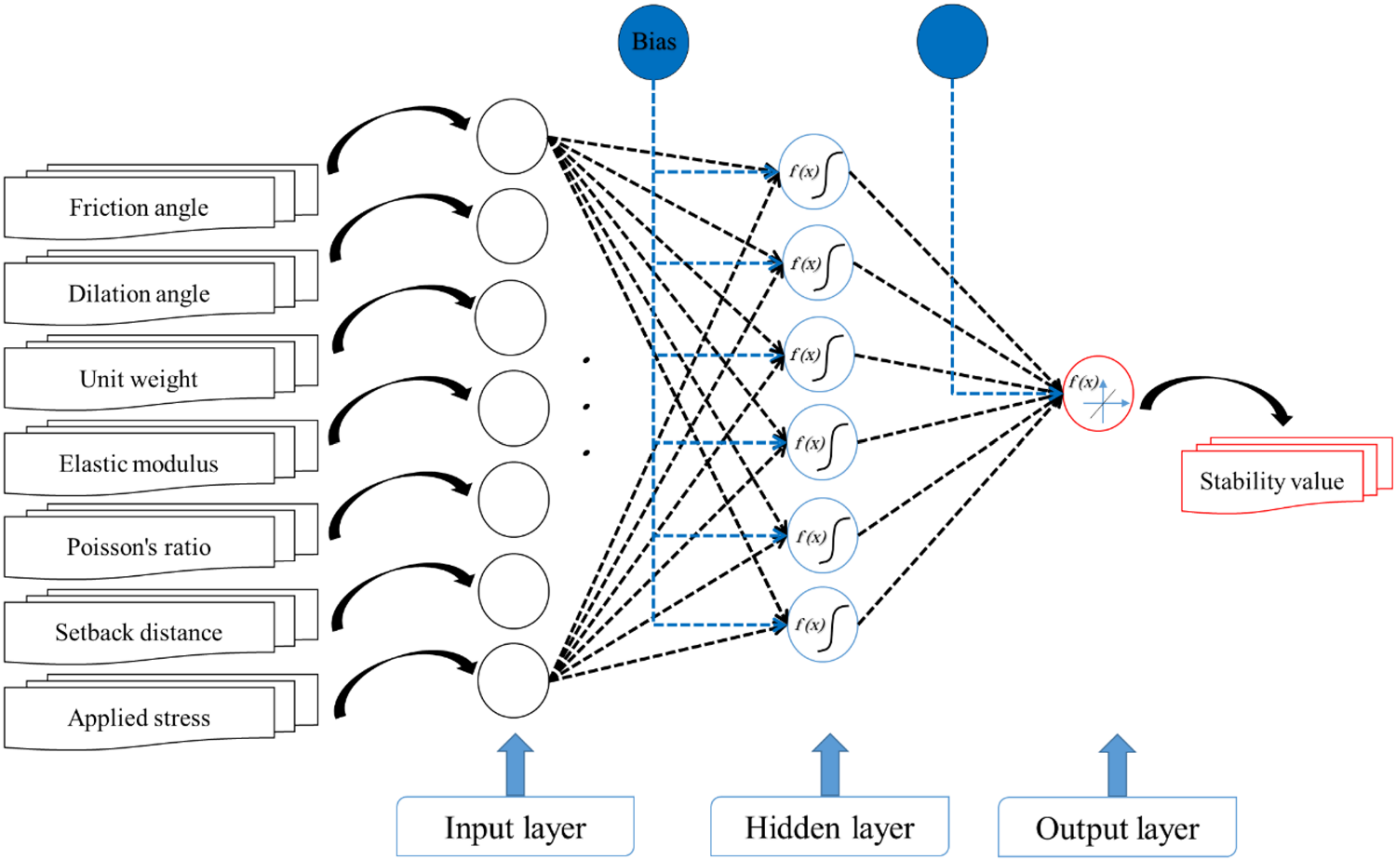
Selected MLP architecture for predicting the settlement.

An MLP neural network, in which the backpropagation (BP)^87^ is used for learning the pattern, represents the basic model. The BP-ANN is composed of one hidden layer. Activation functions for the hidden and output layers are set Tansig and Purelin, respectively. The number of processors in the middle (i.e., hidden) layer is an essential variable in such networks. This parameter is optimized by trying ten different structures (i.e., 7 × x × 1 where x = 1, 2, …, 10). This process revealed that 7 × 6 × 1 is the most reliable structure. This network was subsequently used as the final BP-ANN network (Fig. 2).

### Metaheuristic algorithms synthesized with the ANN

The optimization process is explained in this section. The BSA and EO metaheuristic algorithms are supposed to act as the trainers of the ANN. In this regard, a raw ANN (7 × 6 × 1) and training dataset are considered. The word “raw” means not-trained and the weights and biases of the network (black and blue dashed lines in Fig. 2) are variable that must be determined to attain a trained model. The metaheuristic algorithms are able to find the most promising response to this problem through a repetitive process. As explained, these methods first suggest a random solution and try to minimize the error in several iterations. They use an objective function (OF), that is selected to be root mean square error (RMSE) in this study, to evaluate the goodness of the solution at each iteration. In other words, every response is a matrix of weights and biases that reconstructs the MLP.

**Figure 3:**
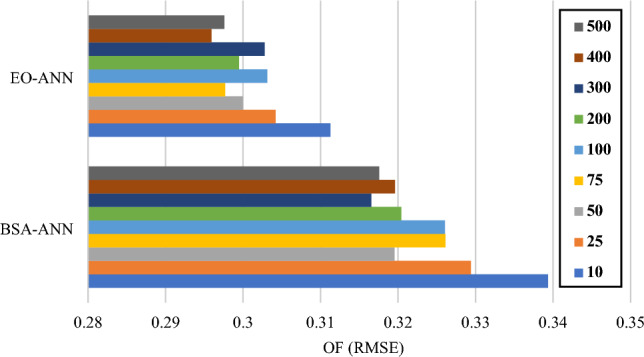
Executed sensitivity analysis based on the population size.

The number of iterations for minimizing the error was set to be 1000 for this work. Figure 3 shows the minimized OF values obtained for different population sizes (i.e., 10, 25, 50, 75, 100, 200, 300, 400 and 500) of the BSA-ANN and EO-ANN ensembles. According to this figure, the OFs of the EO are considerably lower than BSA. A comparison between the tested complexities indicates that the best-fitted population sizes for the BSA-ANN and EO-ANN are 300 and 400, respectively.

**Figure 4:**
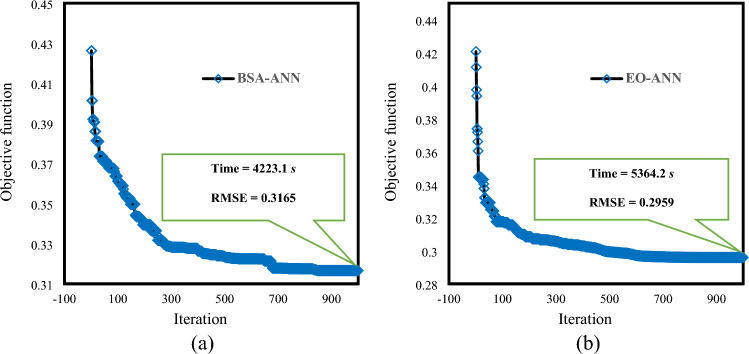
The convergence curves of the best-fitted BSA-ANN and EO-ANN models.

Moreover, the convergence curves of the selected networks are shown in Fig. 4. As is seen, the BSA-ANN and EO-ANN curves start with the RMSEs of 0.42631 and 0.42071 and end up with 0.31656 and 0.2959 at the 1000th iteration. The figures also show that both models have minimized the learning error in the first half of iterations and the curve has remained more or less steady after the 700th iteration.

### Accuracy assessment criteria

The accuracy assessment is done by means of two methods. First, two statistical indices of RMSE and mean absolute error (MAE) are used to measure the error of learning and predictions. Given *Y*_*i observed*_ and *Y*_*i predicted*_ as the expected and predicted SVs, respectively, and also *N* as the number of instances, the formulation of these indices is expressed by the below equations:

15 $$RMSE=\sqrt{\frac{1}{N}\sum_{i=1}^{N}{({Y}_{{i}_{observed} }-{Y}_{{i}_{predicted} })}^{2}}$$

16 $$MAE= \frac{1}{N}\sum_{i=1}^{N}({Y}_{{i}_{observed} }-{Y}_{{i}_{predicted} })$$

Since a classification problem is explored in this work, a well-accepted index called area under the receiving operating characteristics curve (AUROC) is used to measure the accuracy of prediction. Many studies have introduced the ROC curve as a good accuracy indicator in diagnostic issues^88,89^. It can receive values between 0.5 and 1 which indicate poor and ideal predictions, respectively. Given S and F as the total number of stability and failure cases, respectively, Eq. 17 expresses how the AUROC is calculated.

17 $$AUC= \frac{\sum {T}_{P }+ \sum {T}_{N }}{S+F}$$

where T_P_ and T_N_ represent true positive and true negative, respectively.

### Accuracy assessment of the predictive models

Accuracy assessment of the BP-ANN, BSA-ANN, and EO-ANN models is included in two separate parts for the training and testing phases. The learning quality of the ANN (handled by the BP, BSA, and EO methods) is represented by the accuracy of the training phase. Likewise, the capability of the models to generalize the SV pattern is indicated by the testing accuracy. This is because the testing data play the role of stranger conditions to the networks.

**Figure 5:**
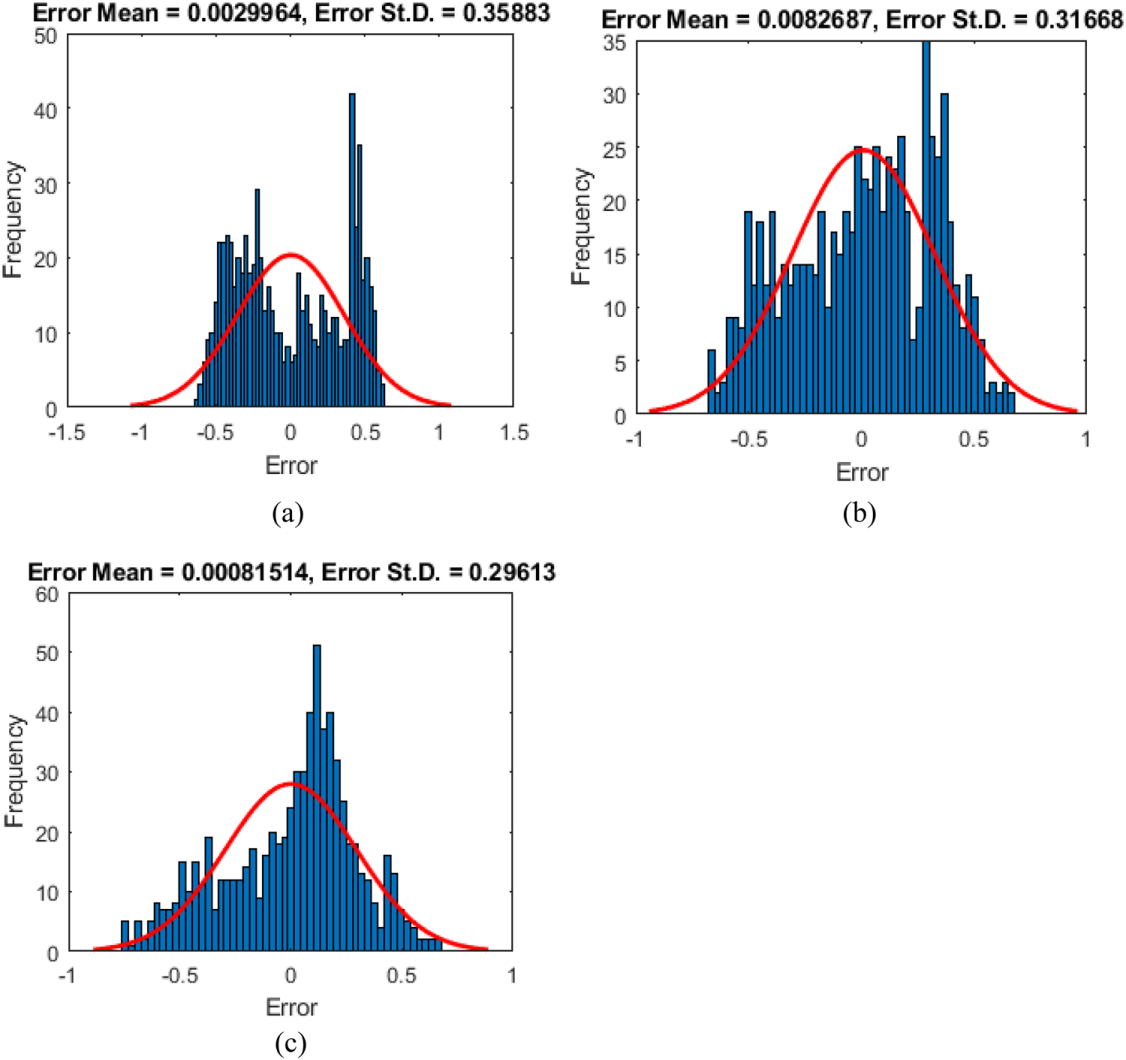
The histogram of the training errors for (**a**) BP-ANN, (**b**) BSA-ANN, and (**c**) EO-ANN.

As mentioned, two error criteria of RMSE and MAE are used. However, the prediction error of each sample is also represented by the difference between the predicted and expected SVs (0 and 1). Figure 5 shows the histogram of the training errors for each model. In this phase, the RMSE of the BP-ANN, BSA-ANN, and EO-ANN models was obtained 0.3585, 0.3165, and 0.2959, respectively. According to these values, the learning process of the hybrid models has been more successful than unreinforced ANN. The same results are obtained for the MAE index. It experienced considerable reductions from 0.3227 to 0.2672 and 0.2397. It denotes that the BSA and EO have trained the ANN more accurately than the BP method.

Moreover, the ROC curves of the BSA-ANN and EO-ANN covered a larger area in comparison with the BP-ANN. The calculated AUROCs showed 95.2, 96.9, and 97.1% accuracy. Pairwise comparison between the obtained ROC curves also confirms a meaningful difference between the performance of the BSA-ANN ~ BP-ANN and EO-ANN ~ BP-ANN. In this sense, p-value was less than 0.0001 for both of these comparisons which means the performances of the considered models are statistically different^90^. This is while the p-value for the comparison BSA-ANN ~ EO-ANN was 0.2997.

**Figure 6:**
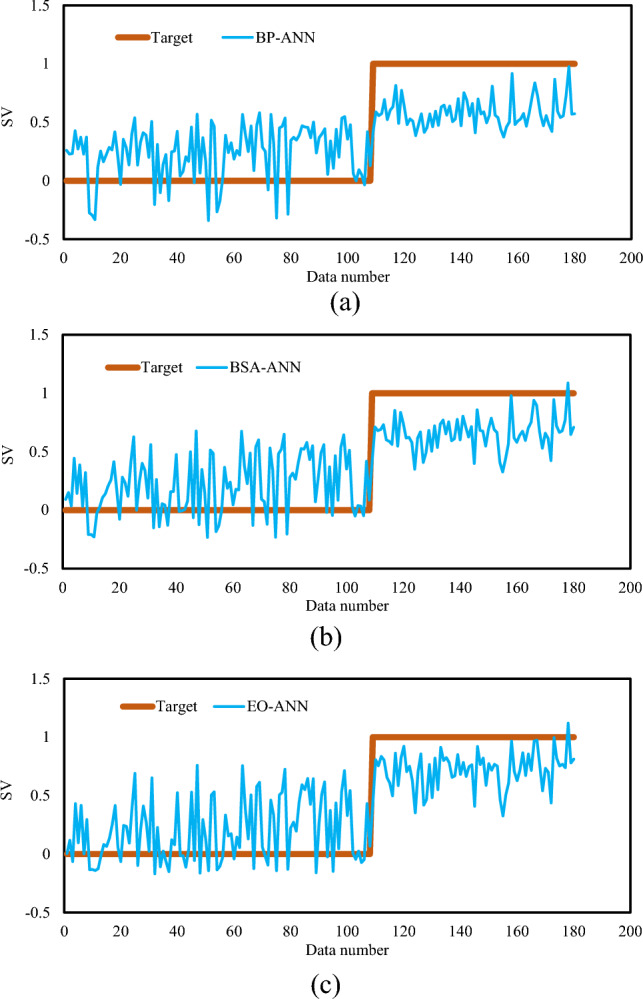
The real SVs versus those obtained by (**a**) BP-ANN, (**b**) BSA-ANN, and (**c**) EO-ANN in the testing phase.

The results of the testing phase are shown in Fig. 6. The responses of the networks are compared with the expected SVs (0 and 1). The maximum and minimum values of real SVs are 1 and 0, while these values range in [− 0.3406, 0.9758], [− 0.2334, 1.0876], and [− 0.1693, 1.1201] for the prediction of the BP-ANN, BSA-ANN, and EO-ANN, respectively.

The RMSE of the typical ANN was 0.3776 which is reduced to 0.3459 and 0.3314 after the incorporation with the BSA and EO metaheuristic optimizers. Also, the MAE values (0.3451 vs. 0.2967 and 0.2687) indicate a higher prediction capability for the ensemble models.

**Figure 7:**
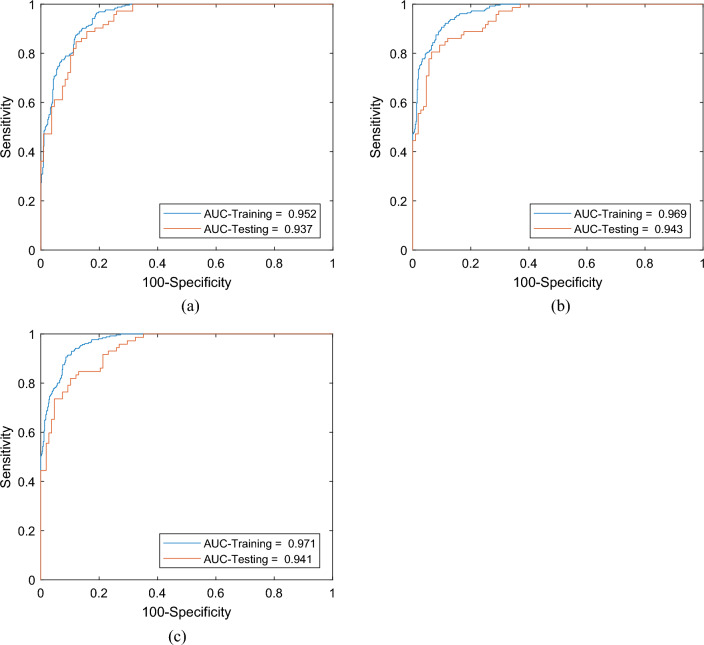
The training and testing ROC curves of the (**a**) BP-ANN, (**b**) BSA-ANN, and (**c**) EO-ANN models (AUC = AUROC).

Figure 7 depicts the ROC curves plotted for evaluating the prediction accuracy of the used models. From these diagrams, it can be seen that all three models have presented a reliable approximation of the stability/failure situation of the soil system. However, the curve of the ensemble models covers a slightly larger area compared to that of BP-ANN (0.937 vs. 0.943 and 0.941).

After confirming the high efficiency of the used BSA and EO algorithms in optimizing the ANN, a comparison between these models outlines the superior optimizer. Referring to all three applied statistical indices (i.e., the RMSE, MAE, and AUROC) in the training phase, the results of the EO-ANN are more promising than BSA-ANN. It means that the EO enjoys more optimization potential. The same results were observed for the testing data in terms of the RMSE and MAE. While the problem of this study was not a regression problem, relevant accuracy indicators (e.g., correlation indices) can also be used for supporting this comparison. Considering respective R^2^ values of 0.4512, 0.5695, and 0.6182 in the training phase and 0.4253, 0.5100, and 0.5439 in the testing phase of the BP-ANN, BSA-ANN, and EO-ANN, it can be said that the outcomes of the EO-ANN are most correlated with target SVs.

### The optimized SV predictive formula

Going back to Sect. “Results and discussion”, Fig. 2 shows the model is structured as 7 × 6 × 1; indicating seven input processors, six hidden processors, and one output processor. There are a total of 55 weights and biases within this network. In this study, these parameters were optimized by two wise metaheuristic techniques. By extracting and arranging these values from the EO-ANN model, the SV predictive formula is produced. Equation 18 expresses this formula as a linear combination of the connecting weights along with one bias term. The parameters α, β, γ, δ, ε, and ζ are the outcomes of the hidden layer that are calculated based on Eq. 19. The weights that connect the input and hidden layer are multiplied by the input parameters (i.e., FA, DA, UW, EM, PR, SD, and AS) added to a bias term. Lastly, the resulting value is affected by an activation function (i.e., Tansig) which is formulated by Eq. 20^91^.

18 $$SV_{EO - ANN} = \, - \, 0.0219 \times \alpha + \, 0.0214 \, \times \beta - \, 0.7746 \, \times \gamma + \, 0.7083 \, \times \delta + \, 0.3176 \, \times \varepsilon - \, 0.9065 \, \times \zeta - \, 0.3080$$

19 $$\left[\begin{array}{c}\alpha \\ \beta \\ \gamma \\ \delta \\ \varepsilon \\ \zeta \end{array}\right]= Tansig\left(\left(\left[\begin{array}{ccccccc}0.5998& 0.5060& -0.8372& -0.9083& -0.3259& 0.9963& 0.1717\\ -0.6825& 0.5284& 0.9527& -0.1774& -0.0922& -0.9864& -0.7776\\ 0.6095& 0.9555& -0.7526& 0.4381& 0.1579& 0.8585& 0.6823\\ 0.6569& 0.9071& -0.0875& 0.4645& 0.7012& 0.4818& -1.0337\\ 0.3572& 0.5213& 0.3003& 0.8042& -0.1631& 0.9038& 1.1361\\ 0.4829& -0.6830& -0.3372& 1.3143& 0.6833& -0.4609& 0.2241\end{array}\right] \left[\begin{array}{c}FA\\ DA\\ UW\\ EM\\ PR\\ SD\\ AS\end{array}\right] \right) + \left[\begin{array}{c}-1.8084\\ 1.0850\\ -0.3617\\ 0.3617\\ 1.0850\\ 1.8084\end{array}\right]\right)$$

20 $$Tansig \,\left(x\right)= \frac{2}{1+ {e}^{-2x}}-1$$

This formula can be directly used to calculate the SV of the studied system. Also, it is believed that similar systems with compatible variables can also benefit from this formula, due to the higher accuracy of the origin model (i.e., EO-ANN). Considering the excellent performance of this model in the testing phase, it was concluded that the EO-ANN model enjoys high generalizability. The reason behind this claim is that the testing data was quite different from the data used for training the model. Therefore, new datasets with compatible conditions would be treated as new testing data. While this formula eliminates the need for sophisticated programming environments, developing a simplified graphical user interface (GUI) such as Excel-based GUIs ^92–94^ would increase its usability.

### More discussion, limitations, and future work

Following the successful application of leading technology for solving various civil and geotechnical problems ^95–99^, this study employed two integrative machine learning models for dealing with the problem of bearing capacity in soil-foundation systems. Many studies can be found in the literature that have shown high accuracy of machine learning algorithms in bearing capacity prediction in various conditions, and accordingly, these models have been recommended for further investigations ^100,101^. In this research, the EO and BSA algorithms could optimize a BP-ANN model and enhance its accuracy of prediction. In the way of accurate predictions, undesirable phenomena such as local minima and overfitting threaten regular machine learning models. Therefore, in studies that employ these models, it may be required to prove the absence of overfitting, local minima, etc^102,103^. However, in the current study, this issue was already avoided, due to the use of powerful optimization algorithms. As per Fig. 4, the solutions of both algorithms have been continuously improved (protected) within a large number of iterations.

Concerning the time-effectiveness of these two algorithms, the computation time taken by the elite models of the BSA and EO (i.e., the population sizes of 300 and 400, respectively) were equal to 4223.2 and 5364.2 s under the same conditions. Thus, while the solution provided by the EO algorithm was more accurate, the BSA could find the global response to the ANN structuring more quickly. Knowing that each algorithm has an advantage, one may select the proper model by considering the importance of both time and accuracy. For instance, in projects where time is a limited source, the BSA can yield a faster (yet reliable) solution. However, engineers are recommended to prefer accuracy for sensitive engineering projects like bearing capacity analysis. The number of involved input parameters is an important task that influences the problem dimension, hence, affecting both optimization time and accuracy. Therefore, optimizing the problem dimension can be effective for dealing with this issue.

**Figure 8:**
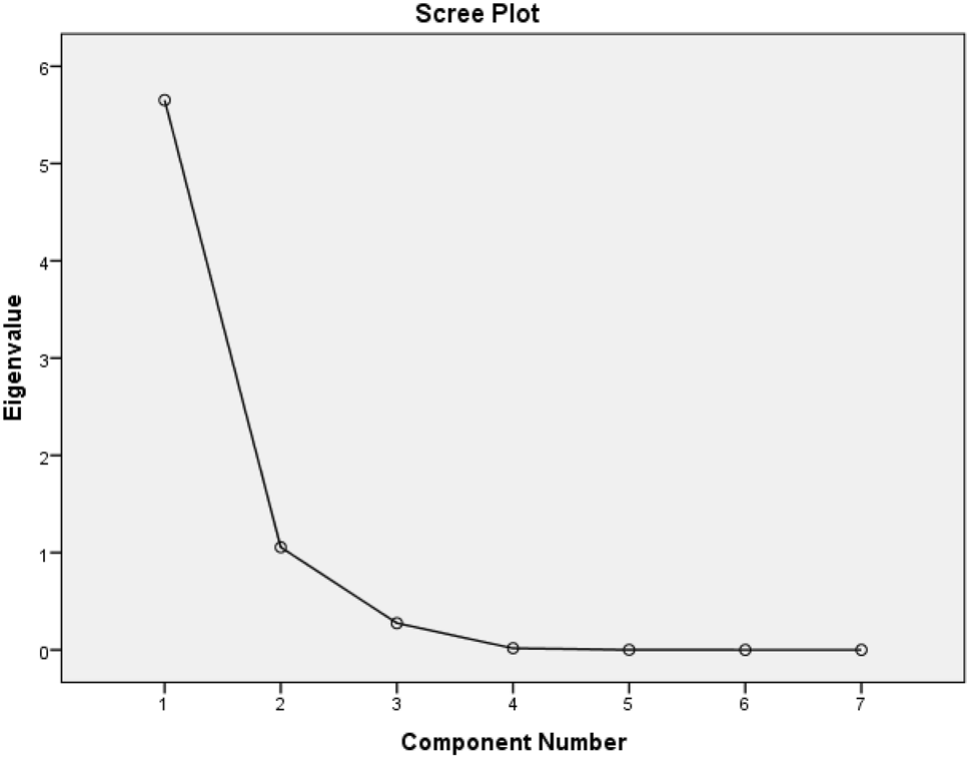
Scree plot of the PCA results.

In addition to Table 2, statistical analysis can be used to show the importance of the input parameters in solving the given bearing capacity problem. The PCA technique^104^ is a popular method for this purpose ^105^. Figure 8 shows the scree plot of the PCs, according to which, two PCs (PC 1 and PC 2) have eigenvalues greater than 1; hence, they are considered significant. These two PCs account for 95.8% variation in the dataset.

**Figure 9:**
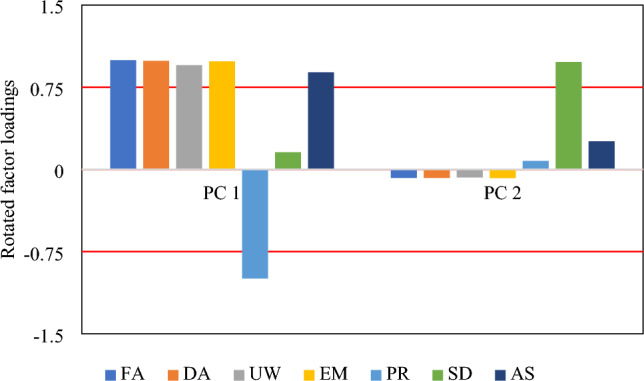
Rotated factor loadings for PC 1 and PC 2.

Figure 9 illustrates the factor loadings obtained for input factors in PC 1 and PC 2. Utilizing the Varimax rotation method and having + 0.75 (and − 0.75) as the significance thresholds^106^, it can be seen that SD is significant in PC 2 while the other six inputs (i.e., FA, DA, UW, EM, PR, and AS) are significant in PC 1. Altogether, all seven input factors are important in the SV prediction of the soil-footing system.

While the focus of the current research was on evaluating the effect of seven input parameters (i.e., FA, DA, UW, EM, PR, SD, and AS), future studies can add more variables in order to develop more comprehensive solutions. For instance, footing size was a constant here, and taking its effect into consideration would result in models which are generalizable to different soil-footing conditions.

The reliability of the used models in this research was assessed and confirmed via three well-known statistical indicators. Two error criteria (i.e., RMSE and MAE) measured the error based on the difference between the expected and predicted SVs and ROC curves were plotted to measure the accuracy. However, there are other indicators (e.g., a20^107^ and error level-cumulative frequency plot^108^) that can be used in the subsequent efforts to support these results.

## Conclusions

Two new metaheuristic algorithms, namely the backtracking search algorithm and equilibrium optimizer were successfully applied to the problem of footing bearing capacity analysis. The BSA-ANN and EO-ANN models were developed to predict the stability value of a system composed of a footing placed on a two-layered soil mass. Comparing the performance of these ensembles with a conventional ANN showed that the training RMSE of the ANN was reduced by 11.72% and 17.46%, respectively. These values were 8.40% and 12.24% for predicting the stability/failure of the studied system. Based on these improvements, the authors can suggest the used models as reliable approaches for the early approximation of the stability situation of similar systems. For more convenient applications, a monolithic predictive formula was also created based on the EO-ANN model. However, referring to the limitations of this research, future studies are recommended to focus on solutions that can (i) improve the applicability of the suggested model/formula in real-world projects, (ii) enhance the efficiency i.e., time-effectiveness, accuracy, and generalizability of the suggested models, (ii) provide comparative assessment of the EO and BSA versus the latest generation of metaheuristic algorithms.

## Data Availability

All data analysed during this study can be shared upon reasonable request from the corresponding author.
